# A novel redox regulator, MnTnBuOE-2-PyP^5+^, enhances normal hematopoietic stem/progenitor cell function

**DOI:** 10.1016/j.redox.2017.02.005

**Published:** 2017-02-10

**Authors:** Y. Zhao, D.W. Carroll, Y. You, L. Chaiswing, R. Wen, I. Batinic-Haberle, S. Bondada, Y. Liang, D.K. St. Clair

**Affiliations:** a Department of Toxicology and Cancer Biology, University of Kentucky, Lexington, KY, USA; b Department of Neurosurgery, University of Texas, Houston, TX, USA; c Department of Radiation Oncology, Duke University School of Medicine, Durham, NC, USA; d Genetic Center, Women and Children's Healthcare, Qingdao, China; e Department of Microbiology and Molecular Genetics, University of Kentucky, Lexington, KY, USA

**Keywords:** HSC, Hematopoietic stem cell, HSPCs, Hematopoietic stem progenitor cells, HPCs, Hematopoietic progenitor cells, ROS, Reactive oxygen species, OXPHOS, Oxidative phosphorylation, SOD, Superoxide dismutase, MnP, MnTnBuOE-2-PyP^5+^, CFU, Colony-forming unit, CAFC, Cobblestone area-forming cell, BMT, Bone marrow transplantations, TF, Transcription factor, OCR, Oxygen consumption rate, ETC, Electron transport chain, BMNCs, Bone marrow nucleated cells, MFI, Mean fluorescence intensity, Nrf2, Nuclear factor (erythroid-derived 2)-like 2, CAT, Catalase, UCP3, mitochondrial uncoupling protein 3, ETS, E twenty-six transcription factors, LSK, Lin^-^, Sca1^+^, c-kit^+^ cells, Lin+, Lineage positive cells, MyPro, Myeloid Progenitor, MnSOD, ROS, Stem, Nrf2, Mitochondrial, ROS

## Abstract

The signaling of reactive oxygen species (ROS) is essential for the maintenance of normal cellular function. However, whether and how ROS regulate stem cells are unclear. Here, we demonstrate that, in transgenic mice expressing the human manganese superoxide dismutase (MnSOD) gene, a scavenger of ROS in mitochondria, the number and function of mouse hematopoietic stem/progenitor cells (HSPC) under physiological conditions are enhanced. Importantly, giving MnTnBuOE-2-PyP^5+^(MnP), a redox- active MnSOD mimetic, to mouse primary bone marrow cells or to C57B/L6 mice significantly enhances the number of HSPCs. Mechanistically, MnP reduces superoxide to hydrogen peroxide, which activates intracellular Nrf2 signaling leading to the induction of antioxidant enzymes, including MnSOD and catalase, and mitochondrial uncoupling protein 3. The results reveal a novel role of ROS signaling in regulating stem cell function, and suggest a possible beneficial effect of MnP in treating pathological bone marrow cell loss and in increasing stem cell population for bone marrow transplantation.

## Highlights

• MnSOD promotes the expansion of normal mouse bone marrow stem cells.
• MnP enhances mouse stem cell in number and function.
• MnP reduces mitochondrial function in mouse bone marrow cell.
• MnP induces antioxidant defense by elevating Nrf2 and ETS transcription activities.

## 1 Introduction

Hematopoietic stem cells (HSCs) have the capacity for self-renewal and multilineage differentiation. It is well-documented that HSCs reside in a hypoxia niche [1] and have a low level of reactive oxygen species (ROS) compared with their mature progeny [2], [3]. Hypoxia plays critical roles in maintaining stem cell quiescence and stemness [4], [5]. Life-long measurements of local oxygen tension (*pO*_*2*_) in the bone marrow of living mice show that the absolute *pO*_*2*_ of bone marrow is <32 mm Hg and that the lowest *pO*_*2*_ in the deeper peri-sinusoidal regions where HSCs reside is only 9.9 mm Hg [6]. In adult stem cells such as hematopoietic stem cells or mesenchymal stem cells, hypoxia prolongs the lifespan of stem cells, increases their self-renewal capacity, and reduces differentiation in culture [3], [7]. Culturing bone marrow cells with 1–3% O_2_ enhances HSCs expansion and engraftment compared to the 21% O_2_ counterparts [8], [9].

The roles of mitochondria and reactive oxygen species (ROS) in regulating stem cell fate are crucial and complex. It is generally thought that stem cell self-renewal relies primarily on glycolysis and the pentose phosphate pathway, and also on a deliberate suppression of oxidative phosphorylation (OXPHOS) [10]. Some of the experimental evidence in support of this concept includes: 1) Direct measurement of the incorporation of ^13^C from glucose into lactate indicates that long term hematopoietic stem cells (LT-HSCs) rely on anaerobic glycolysis, and have lower rates of oxygen consumption and lower ATP levels than other cells in bone marrow [11]; 2) Forced activation of OXPHOS leads to loss of stem cell properties and increased differentiation and apoptosis [12]; 3) Inhibition of complex III of the mitochondrial respiratory chain using antimycin A or myxothiazol promotes human ESC self-renewal and pluripotency [13]; 4) Genetic ablation of Hypoxia-inducible factors (HIFs), which causes an increase in ROS and activation of OXPHOS, results in the loss of quiescence and the self-renewal properties of hematopoietic stem cells (HSCs) [14]; 5) c-kit-positive stem/progenitor cells show lower basic levels and faster clearance of accumulated intracellular ROS, and higher resistance to oxidative stress compared to c-kit-negative mature mononuclear cells [15]. However, whether and how the subtle changes in mitochondrial function and ROS production modulate stem cell function and survival remain unknown.

Mitochondria are the primary site of superoxide radical generation. The superoxide dismutase (SOD) family of enzymes catalyzes the dismutation of superoxide anion (O2^•-^) radical to hydrogen peroxide (H_2_O_2_) and molecular oxygen (O_2_). This family of enzymes is comprised of MnSOD, located in the mitochondrial matrix, and Cu, ZnSOD, located in the mitochondrial intermembrane space, cytosol and extracellular space. The presence of MnSOD is essential for the survival of all aerobic organisms from bacteria to humans [16], [17]. Since MnSOD has a critical role in controlling ROS generated in mitochondria, we examined the effect of MnSOD on hemapoietic stem and progenitor cells (HSPCs) in transgenic mice expressing the human MnSOD gene. We found that overexpressing MnSOD in the mitochondria of transgenic mice enlarges the pool of HSPCs compared to the result for wild-type littermates. To further explore the impact of ROS on bone marrow cells, we tested a synthetic compound, Mn(III) *meso*-tetrakis(*N*-*n*-butoxyethyl-pyridinium-2yl)porphyrin, MnTnBuOE-2-PyP^5+^, BMX-001 (MnP), an MnSOD mimetic that catalyzes superoxide dismutation in a SOD-like manner. The chemistry and biology of the family of Mn-containing porphyrin have been reviewed extensively [18]. The protective effects of SOD mimetics in the normal cells of a number of animal models have been reported. For instance, in normal tissues of animal models, MnP and an earlier analog, MnTE-2-PyP^5+^(AEOL10113, BMX-010), were found to be effective radioprotectants of radiation-induced injuries of salivary gland and mouth mucosa [19], bone marrow [20], brain [21], rectum [22] and urogenital tissues [23]. Consequently, several SOD mimetics are being tested in Phase I/II clinical trials for protection of normal tissues against injury from ROS-generating therapeutics including ionizing radiation.

MnP, the newest generation of SOD mimetic, is optimized for catalytic potency and safety/toxicity [24]. It is preferentially localized in the mitochondria and has been shown to have very limited side effects in animal models [18]. In the present study, we report that MnP treatment of freshly isolated mouse bone marrow cells and mice increases the number of bone marrow HSPC cells and improves the function of long-term engraftment and multi-lineage differentiation of the HSCs. The results reveal a novel aspect of ROS regulation in stem cell function and strengthen the clinical potential of MnP.

## 2 Materials and methods

### 2.1 Reagents, treatment and animal study

MnP was synthesized and characterized by Dr. Ines Batinic-Haberle and her colleagues (Duke University School of Medicine, Durham, NC, USA). *In vitro* treatment of MnP was done on freshly isolated bone marrow cells from 9 to 12 weeks-old C57BL/6 female mice with either H_2_O (2–5 μl/ml of culture media as vehicle depending on the concentration of MnP used) or 5–20 μM of MnP for 1–16 h at 37 °C in 5% O_2_ incubator. *In vivo* treatment was performed using in-house bred, 9–12 weeks-old, female C57BL/6 mice. The mice were treated with either saline (vehicle) or MnP at 2 mg/kg, 3 times/week subcutaneously (s.c.) for up to 60 days. All animal studies were conducted using procedures approved by Institutional Animal Care in accordance with the NIH Guide for the Care and Use of Laboratory Animals.

### 2.2 Immunofluorescent staining of bone marrow cells

Bone marrow cell isolation, immune-staining and flow cytometry were performed as described [25]. In brief, cells were extracted from two femurs and tibias of mouse. RBCs were lysed to get bone marrow nucleated cells (BMNCs). The BMNCs were stained with the following antibodies: Biotin-conjugated lineage markers, including CD5 (Cat# 553019), CD8a (Cat# 553029), CD45R/B220 (Cat# 553086), CD11b/Mac-1 (Cat# 553309), Ly-6G/Gr-1 (Cat# 553125), TER119/Ly-76 (Cat# 553672) followed by Streptavidin-conjugated secondary fluorescent antibody (Cat# 554063) and stem cell–specific markers, PE or PEcy7-conjugated Ly-6A/E (Cat# 553336, 558162) and APC or Percp Cy5.5-conjugated CD117 (Cat# 553356, 560557). All antibodies were purchased from Pharmingen. Flow cytometric analysis was performed on FACS Calibur flow cytometer (Becton Dickinson, San Jose, CA, USA).

### 2.3 Colony-forming cell assay (CFC) and cobblestone area-forming cell (CAFC) assays

The CFC assay was described previously [25]. Briefly, freshly isolated total bone marrow cells were suspended in Methocult GF M3434 methylcellulose medium (StemCell Technologies, Vancouver, B.C., Canada). Triplicate cultures were set up for each specimen according to the manufacturer's instructions. After 14 days of culture, the total colony- forming unit (CFU) were counted.

The CAFC assay was described previously [25]. Briefly, a monolayer of FBMD-1 stromal cells was plated in 96-well plates and grown to confluent; whole bone marrow cells were seeded at 81,000, 27,000, 9000, 3000, 1000 or 333 cells per well in CAFC medium (Iscove's MDM supplemented with 20% horse serum, 10^−5^ M hydrocortisone, 10^−5^ M 2-mercaptoethanol, 100 U/ml penicillin, and 100 μg/ml streptomycin). Twenty replicate wells per cell number were counted. The frequency of CAFC was determined on days 7, 14, 21, 28 and 35. Wells were scored positive if at least one phase dark hematopoietic clone (containing five or more cells) was seen. The frequencies of CAFCs were calculated using the L-Calc program (StemCell Technologies).

### 2.4 Serial bone marrow transplantations (BMT)

One million bone marrow cells (CD45.2) treated either with 20 μM MnP for 16 h *in vitro* or with MnP *in vivo* (2 mg/kg, 3 times/week s.c. for 60 days) were mixed with 10^6^ of helper cells (CD45.1) in 200ul PBS, and the mix was injected intravenously into lethally irradiated (9 Gy) CD45.1 recipient mice (n=6 for each control or MnP treatment). At 16-week post the first BMT, total bone marrow cells were isolated and analyzed for CD45.2 positive stem and progenitor cell frequency, and 10×10^6^ cells/mouse were transplanted to another CD45.1 mouse (n=6) as secondary BMT. The third BMT was performed with 10×10^6^ cells/mouse of total bone marrow cells from the secondary bone marrow transplanted mouse at week 16 post-secondary BMT (n=6 for treatment and control). CD45.2 positive T cell, B cell, granulocytes and macrophages of peripheral blood were analyzed every four weeks post each BMT to check transplanted cell engraftment as described previously [25]. Peripheral blood cell counts were performed at the end of each 16-week BMT.

### 2.5 Quantitative real-time PCR (qPCR)

Total RNA was isolated from approximately 5×10^4^ LSK (lineage negative, c-kit and Sca1 positive) cells using RNeasy Micro Kit (Qiagen, Cat# 74004). First-strand cDNA was reverse-transcribed and then preamplified with the Transcriptor first strand cDNA synthesis kit and cDNA Pre-Amp Master mix (Roche, Cat# 04379012001, 06720455001), respectively. Pre-amplification for the tested genes was 12 cycles. qPCR was performed with LightCycler 480 Probes Master system (Roche, Cat# 04707494001). All experiments were performed following manufacturers’ instructions. Actin and 28 S were used as endogenous controls for normalization of gene expression.

### 2.6 Mitochondrial function and ATP production measurement

Oxygen consumption in cells was determined using the Seahorse Extracellular Flux (XF-96) analyzer (Seahorse Bioscience, Chicopee, MA, USA). Bone marrow cells were isolated and treated with MnP at 20 μM for 2 h. The cells were then harvested, antibody-labeled and flow cytometry sorted in ice or at 4 °C. 200,000 LSK cells in triplicate for treatment or control were cytospinned to the bottom of a XF96 Tissue Culture Plate (Seahorse Bioscience) coated with BD Cell-Tak Cell Adhesive. OCR was measured three times and plotted as a function of cells under the basal condition followed by the sequential addition of oligomycin (1 μg/ml), FCCP (1 μM), or antimycin (2 μM) and rotenone (1 μM) for the ATP-linked, maximum, and reserve capacities of oxygen consumption, respectively.

BMNCs were isolated from mice (n=6). The cells were treated with either vehicle or 20 μM MnP for 1 h. at 37 °C, stained and sorted for LSK and MyPro (Lineage negative, c-kit positive, Sca1 negative) cells in ice or at 4 °C. Sorted cells (3×10^4^) were lysed, and intracellular ATP activity was measured using the Luciferase ATP Bioluminescent somatic cell assay kit (Sigma, Cat# FLASC) following the manufacturer's instructions.

### 2.7 Dichlorofluorescein (DCF) and GSH/GSSH analyses

DCF assays were performed as previously described [26]. Briefly, after treatment, BMNCs were immune-stained and incubated with 10 μg/ml of carboxy-H2DCFDA (sensitive to oxidation, Invitrogen) or 1 μg/ml of oxidized carboxy-DCFDA (DCFDA, insensitive to oxidation, Invitrogen) in PBS /0.5% FBS at 37 °C for 20 min. The fluorescence in cells preloaded with carboxy-H2DCFDA was normalized to that in cells preloaded with DCFDA (ratio of H2DCFDA/DCFDA) to control for the cell number and dye uptake. The ester cleavage differences between different treatment groups were measured.

Sample extraction and LC-MS/MS analysis of GSH and GSSG have been previously described [27]. In brief, BMNCs treated with either vehicle or MnP were centrifuged at 1300 rpm for 5 min. Cells were resuspended in 75 μl of extraction buffer (2% TCA, 1 mM EDTA), incubated on ice for 15 min, then vortexed for 30 s and incubated on ice for an additional 15 min. Cellular debris was then pelleted by centrifugation at 4000×*g* for 10 min. Sample extracts were then collected and pH was adjusted to 2.0 prior to LC-MS/MS analysis as previously described [27].

### 2.8 Transcription factor (TF) profiling and western blots

BMNCs were obtained from C57B/L6 mice (5 mice/group). Cells were treated with either vehicle or 20 μM MnP for 1 h at 37 °C, immunostained and flow cytometry sorted for lineage marker negative (Lin-) and CD117 (ckit) positive cells (LK) at 4 °C. Nuclear extractions were isolated with Nuclear extraction kit by Signosis (Cat SK-0001) following manufacturer's protocol. TF profiling was performed using the Oxidative stress TF activation profiling plate array (Signosis Cat# FA-1005) by following the manufacturer's instructions. Triplicate wells with the nuclear extracts from 3 groups of mice were used for the TF profiling.

Western blots were prepared using the same protocol as described previously [26]. Briefly, BMNCs were treated with vehicle or 20 μM of MnP for 16 h at 37 °C, harvested, stained and flow cytometry sorted for LK cells. Ten micrograms of total protein from each cell lysate were loaded on SDS-PAGE and blotted with various antibodies.

### 2.9 Statistical analysis

The TF profiling was done at n=3 group of 5 mice. Other experiments were done with samples n≥6. The experiments were conducted at least three times to verify the reproducibility of the findings except for the TF profiling and serial BMT. Statistical analyses were carried out with Statistical Analysis System software (SAS Institute, Cary, NC, USA) and P values were calculated using the Student's *t*-test.

## 3 Results

### 3.1 MnP promotes the expansion of mouse bone marrow HSPCs

**Fig. 1:**
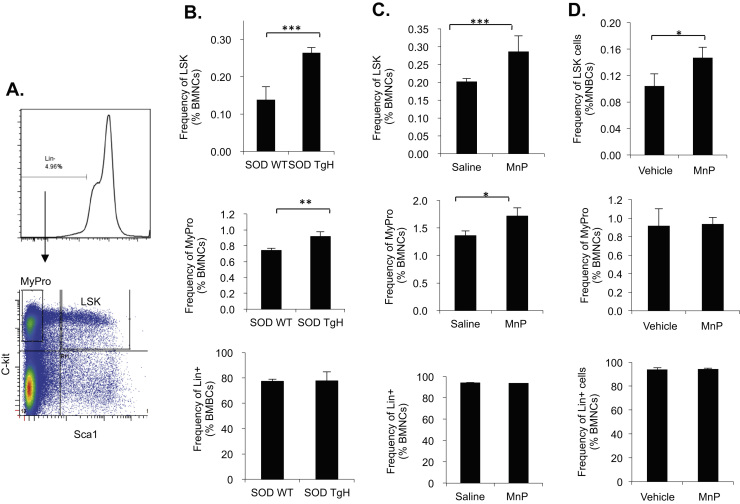
LSK & MyPro cell frequency is increased in MnSOD transgenic mice and the cells treated with MnP *in vitro* and *in vivo*. A. The gating strategy of flow cytometry analysis. B. Bone marrow cells were isolated from wild- type (SOD WT) or MnSOD transgenic (SODTgH) mice. C. C57B/L6 mice (n=8) were injected (i.c.) with vehicle (Saline) or MnP (MnP) at 2 mg/kg, 3 times/week for 60 days. D. Freshly isolated bone marrow cells from C57B/L6 mice (n>6) were treated with vehicle (H_2_O) or 20 μM MnP (MnP) for 16 h *in vitro*. Cells were immunostained and flow cytometry analyzed. Upper panels are the percentage of LSK cells (lin^-^ Sca1^+^ c-kit^+^) in the total bone marrow nucleated cells (BMNCs). The middle and lower panels are MyPro (lin^-^ Sca1^-^ c-kit^+^) and lineage positive (Lin+) cells, respectively. *P<0.05 was considered significant (Student's *t*-test). *P<0.05; **P<0.01; ***P<0.001 as compared with controls for this and the following figures. Data represent the mean+SD of at least three independent experiments.

We have previously generated transgenic mice expressing the human MnSOD gene and performed extensive characterization of the animals [28]. We have verified that MnSOD is located in the matrix of mitochondria and that there is no significant change in other antioxidant enzymes or small molecular weight antioxidants that may impact the cellular redox state. Bone marrow cell frequency analysis of transgenic mice expressing human MnSOD shows that the LSK cells [lineage negative (Lin-) / Ly-6A/E positive (Sca1+) / c-kit positive (c-kit+)] bone marrow stem/progenitor cells and myeloid progenitor cells (MyPro) (Lin-, c-kit+ and Scal1-) were significantly increased by ~40% and 18% respectively compared to the wild-type (wt) littermates (Fig. 1B upper and middle panels), whereas the Lin+ mature cells have no change (Fig. 1B lower panel). We examined the effect of MnP on bone marrow cells since MnP is designed to especially mimic MnSOD. We treated freshly isolated mouse BMNCs with 5, 10, 20 or 25 μM of MnP for 16 h under 5% O_2_ culturing conditions. The results show that at 10–25 μM, MnP significantly increased LSK frequency (data not shown). At 20 μM MnP, the increase of LSK was about ~30%, but there was no significant influence on MyPro and Lin+ cells (Fig. 1D upper, middle and lower panels, respectively). To determine if treatment with MnP *in vivo* could also increase LSK population in mouse bone marrow, we injected MnP at 2 mg/kg s.c., 3 times/week into C57B/L6 mice for 60 days. The results show that MnP given *in vivo* increased LSK cells by ~30% and MyPro by ~20% (Fig. 1C upper and middle panels), without any significant effect on Lin+ cells (Fig. 1C lower panel). We additionally performed peripheral blood counts on the 60-day MnP-treated mice and the wild- type and MnSOD transgenic mice. The peripheral blood counts show no significant change (data not shown). Thus, MnP or overexpressing MnSOD in mice enlarges the HSPC pool of bone marrow without altering the mature cell population, suggesting that mature cells are tightly regulated under normal physiological conditions.

**Fig. 2:**
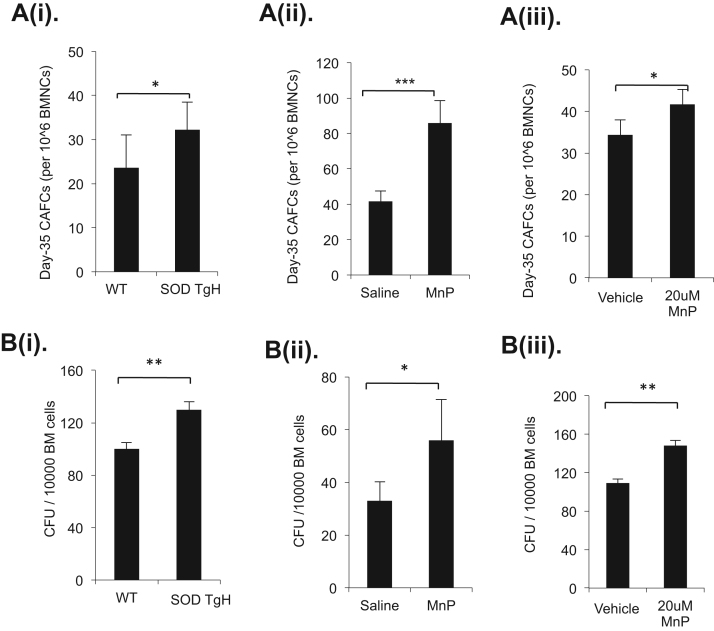
MnP promotes clonogenic function of HSCs and HSPCs. A. The clonogenic function of HSCs was measured at day 35 with CAFC assay. B. The HSPC clonogenic function was determined by counting the total colony forming units per 10,000 BMNCs in CFC assay at day 14. (i) Cells isolated from MnSOD wild- type (WT) and transgenic mice (SOD TgH). (ii) MnP 60-day *in vivo* treatment. (iii) MnP 16 h *in vitro* treatment. (n>=3). Data represent the mean+SD of three independent experiments.

To further examine the functional change of the increased stem and progenitor cells after MnP treatment, we performed CAFC and CFU assays, which measure HSC and progenitor clonogenic functions, respectively. At day 35 of CAFC assays, the approximate increases of cobble stone colonies for the bone marrow cells from MnSOD transgenic mice compared to wild- type controls and for MnP *in vivo-* and *in vitro-* treated bone marrow cells were 30%, 50% and 18% respectively (Fig. 2A, i, ii, iii). The results suggest that bone marrow stem cells maintain normal or increased clonogenic function. Using a MethoCult-based CFC assay, we tested the progenitor cell colony forming function. The total colony forming units (CFU) at day 14 were increased by about 23%, 45% and 18% for the bone marrow cells from MnSOD transgenic mice and MnP in *in vivo-* and *in vitro-* treated compare to controls, respectively (Fig. 2B, i, ii, iii). The results are consistent with the slightly increased frequency of MyPro cells seen from Flow cytometry analysis in the 60-day MnP-treated and MnSOD transgenic mice. Interestingly, the total CFUs were also increased in the 16-hr *in vitro* MnP- treated cells. The results suggest a normal or enhanced function of the progenitor cells.

### 3.2 MnP enhances long-term stem cell engraftment and multilineage differentiation after serial bone marrow transplantation (BMT)

**Fig. 3:**
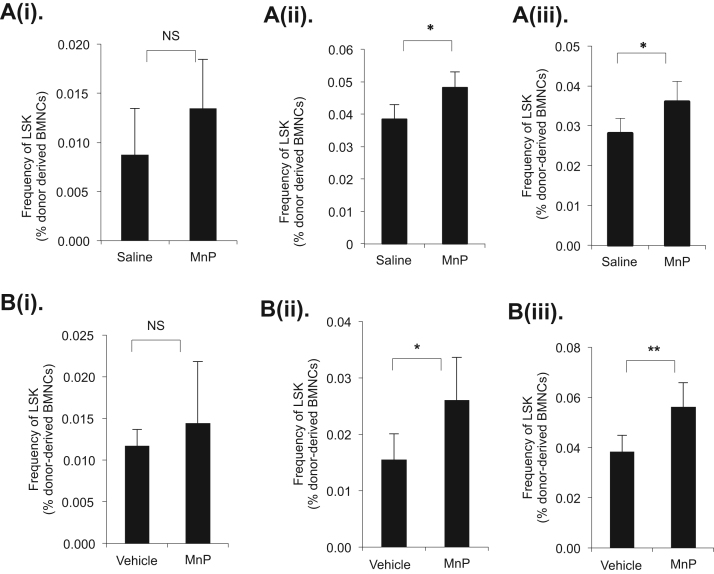
MnP increases HSC engraftment and multilineage differentiation by serial bone marrow transplantations (BMT). A. Donor cells were isolated from 60-day saline or MnP *in vivo* treated mice (n=6 for vehicle or MnP treatment). B. The donor cells were *in vitro* treated with vehicle or 20 μM MnP (MnP) for 16 h (n=6 vehicle or MnP treatment). Percentage of LSK cells of the donor-derived (Ly5.2) bone marrow nucleated cells (BMNCs) in the recipients (n=6 for each treatment) was analyzed by flow cytometry. (i) The results at 16 weeks post the first BMT. (ii) The results at 16 weeks post the second BMT. (iii) The results at 16 weeks post the third BMT. Data represent the mean+SD.

The purpose of serial BMT is to test the self-renewal and multilineage differentiation capabilities of HSCs. To examine the long-term engraftment and repopulation capacity of stem cells after treatment with MnP, we performed three rounds of serial bone marrow transplantations. 10^6^ of total bone marrow cells (CD45.2) treated with either vehicle or MnP *in vitro* for 16 h or *in vivo* for 60 days and 10^6^ of helper cells (CD45.1) were used as donor cells. The donor cells were bone marrow transplanted to lethally irradiated recipient mice (CD45.1). Peripheral blood samples were collected and analyzed four times, at four-week intervals, for the donor cell engraftment. Animals were euthanized at 16 weeks, and the donor- derived (CD45.2) peripheral blood as well as bone marrow cells were analyzed by flow cytometry. At the same time, 10^7^ total bone marrow cells from the first or second transplanted animal were used for the second or the third BMT, respectively. Three rounds of BMT at 16- week intervals (for a total of 48 weeks) were performed. Our results show that the LSK cells slightly increased at 16 weeks post the first BMT with no significance (Fig. 3A (i) and B (i)), and increased by 20% and 17% respectively when *in vivo-* treated BM cells were used as donor cells at the end of the second and the third BMTs (Fig. 3A (ii), (iii)). When using *in vitro* treated bone marrow cells as donors the increases were 39% at the end of second BMT and 33% at the end of third BMT respectively (Fig. 3B (ii), (iii)). We also flow-cytometry analyzed CD45.2 positive T-cell, B-cell and Granulocytes from bone marrow of each BMT at the end of 16 weeks and found no significant difference between control and treated cells. The results at 48 weeks are shown in Supplemental Fig. 1. Peripheral blood counts every four weeks during the three rounds of BMT show no significant change compared to the BMT with control-treated cells (data not shown) suggesting that MnP can specifically increase HSC function without affecting downstream progenies. Since long-term engraftment and multilineage development are the gold standard of stem cell function, the results suggest that MnP enhances the function of bone marrow stem cells.

### 3.3 MnP reduces mitochondrial oxidative phosphorylation and intracellular ATP production in bone marrow cells

**Fig. 4:**
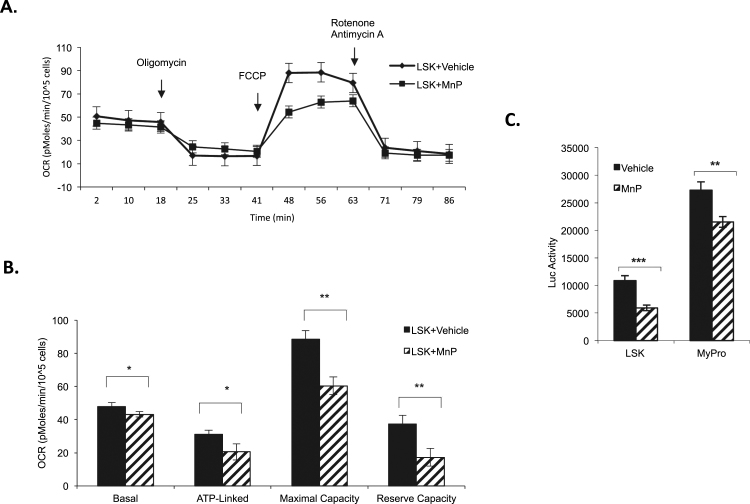
MnP inhibits mitochondrial oxidative phosphorylation and ATP production in LSK. A. The raw data of oxygen consumption rate (OCR) of Seahorse analysis on LSK cells. LSK cells were isolated and treated with vehicle or 20 μM MnP (MnP) for 2 h at 37 °C in 5% O_2_ incubator. The cells were then harvested, immunostained, flow cytometry sorted, and seahorse analyzed in the presence of the mitochondrial inhibitors (Oligomycin, 1 μg/ml, FCCP 1 μM, or Rotonone 1 µM and Antimycin 2 µM). B. The quantitation of basal, ATP-linked, the maximum, and the reserve capacity of oxygen consumption rate of panel A (n=3). C. The freshly isolated bone marrow cells were treated with either vehicle or 20 μM MnP (MnP) for 1 h, immune-stained and sorted. Equal amounts of cell lysate of LSK and MyPro cells were analyzed for ATP production cells (n>6). Data represent the mean+SD of at least three independent experiments.

Stem cells rely primarily on glycolysis and the pentose phosphate pathway, with a deliberate suppression of oxidative phosphorylation (OXPHOS) [10] for their energy demand and maintenance of stemness. Since the cellular localization of MnP is mainly in the mitochondria, we next checked the function of mitochondria and the energy production in bone marrow cells after MnP treatment. Freshly isolated BMNCs were treated with vehicle or 20 μM MnP in 5% O_2_ incubator for 2 h. LSK cells were flow cytometry sorted at <4 °C. Triplicate 200,000 LSK cells were cytospinned to the bottom of the Seahorse microplate, and a Seahorse XF analyzer was used to measure oxygen consumption rate (OCR). Fig. 4A and B indicates that basal OCR, a measure of total oxygen consumption at basal conditions, was reduced by 10% in LSK cells after MnP treatment. Oligomycin inhibits ATP synthase in complex V in the electron transport chain (ETC). Upon Oligomycin application, the ATP linked that represents the oxygen consumption linked to ATP synthesis through OXPHOS was reduced by 35%. The injection of FCCP decouples ATP synthesis and hydrogen ion transport in ETC, leading to rapid consumption of energy without the generation of ATP, and is used to measure the maximal oxygen consumption capacity of mitochondria under stress conditions. We observed that MnP treatment reduced the maximal capacity by 33% in LSK cells. Rotenone and antimycin are used to inhibit the function of complexes I and III, respectively, leading to complete inhibition of ETC. The reserve capacity is calculated as the difference between maximal OCR and basal OCR. Our result shows that the mitochondrial reserve capacity was reduced by nearly 44% after MnP treatment (Fig. 4A & B). Thus, MnP-treated bone marrow HSPC cells, at early time, have a reduced level of mitochondrial oxygen consumption, which suggests reduction of mitochondrial function and is consistent with an enhanced stem cell population.

To further examine the metabolic change caused by MnP treatment, we measured total cellular ATP levels by using a Luciferase ATP Bioluminescent somatic cell assay kit. To observe early changes, mouse BMNCs were isolated, treated with vehicle or 20 μM MnP in a 5% O_2_ incubator for 1 h. The intracellular ATP level in cell lysate from 3×10^4^ LSK or MyPro cells were determined with multiple repetitions. As shown in Fig. 4C, the total ATP level was reduced by 45% in LSK cells and 20% in MyPro cells, which is consistent with the reduction of OXPHOS. Collectively, MnP directly reduced mitochondrial OXPHOS function in HSPCs, and these results are consistent with the notion that the reduction of mitochondrial OXPHOS and ATP production facilitates the maintenance of stem cell pool and function. Additionally, the difference of ATP reduction in more primitive LSK compare to MyPro caused by MnP treatment suggests that either LSKs are more sensitive to MnP or have stronger mechanism to reduce their mitochondrial function.

### 3.4 MnP acts as a mild pro-oxidant but does not affect redox homeostasis in HSPCs

**Fig. 5:**
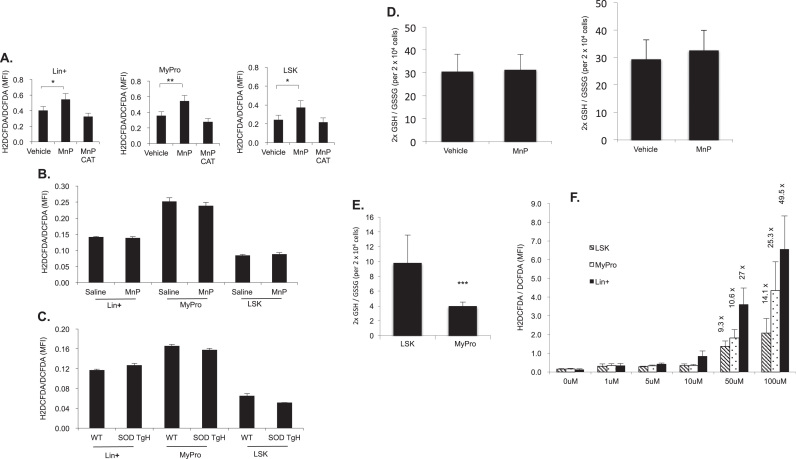
ROS status in different lineage cells physiologically and after treatment with vehicle or MnP or H_2_O_2_. A. BMNCs were isolated and treated *in vitro* with vehicle or 20 μM MnP (MnP) or 20 μM MnP+Catalase (CAT) at 4000U/ml/100,000 cells for 2 h at 37 °C. The cells were immunostained at 4 °C, incubated with H2DCFDA, DCFDA or H2DCFDA +/- CAT, for 15 min at 37 °C and flow cytometry analyzed. The mean fluorescence intensities (MFIs) in Lin+, MyPro and LSK cells are presented (n>6). B. BMNCs were isolated from 60-day saline or MnP treated mice (n=8) and incubated with H2DCFDA or DCFDA. C. BMNCs were isolated from wild- type (WT) or MnSOD_2_ transgenic mice (SOD TgH) (n=6). D. Freshly isolated BMNCs were treated *in vitro* with vehicle or MnP for 2 h (left panel) or 16 h (right panel). 2×10^4^ of LSKs were HPLC analyzed. The ratios of 2xGSH/GSSG are presented. E. The ratio of 2xGSH/GSSG on the freshly isolated LSK or MyPro cells. F·H_2_O_2_ treatment of LSK, MyPro and Lin+ cells. The BMNCs were isolated, then incubated with H2DCFDA or DCFDA for 20 min and with different concentrations of H_2_O_2_ for 15 min at 37 °C, washed and flow cytometry analyzed. The fold changes labeled above the bars are calculated *vs.* the 0 μM H_2_O_2_ treatments. Data are represented as the means+SD.

It is generally thought that elevated ROS favors stem cell differentiation and reduces cell quiescence. MnP, as an MnSOD mimetic, is developed to scavenge O_2_^•−^. Therefore, we next examined the intracellular ROS level of the freshly isolated BMNCs after incubation with 20 μM MnP or vehicle for 2 h. The cells were immunostained at 4 °C and labeled with 10 μg/ml of carboxy-H2DCFDA (sensitive to oxidation) or 1 μg/ml of DCFDA (insensitive to oxidation) for 20 min at 37 °C. Surprisingly, we found that the normalized DCF fluorescence, expressed as mean fluorescence intensity (MFI), in the MnP-treated Lin+, MyPro and LSK cells was elevated by 26%, 33% and 35%, respectively, compared to the vehicle treatment (Fig. 5A). The addition of catalase (CAT) completely inhibited the increase of the DCF signal suggesting that the elevated ROS is superoxide-derived H_2_O_2_. We also examined ROS status in the BM cells from 60-day treated mice and MnSOD transgenic mice. The H2DCFDA/DCFDA level in three cell types has no significant difference between saline and MnP treatments, and MnSOD transgenic and wild- type mice (Fig. 5B & C). Based on these results we hypothesized that MnP might act as a mild pro-oxidant elevating H_2_O_2_ in bone marrow cells. The ROS elevation only occurred at the 2 h *in vitro* treatment and not in the *in vivo* treatment suggesting that the pro-oxidative effect of MnP is transient and might be overcome by adaptive antioxidative responses.

Acting as antioxidants as well as potential pro-oxidant generators, SOD mimetics convert O2^•-^ to H_2_O_2_. Thus, a SOD mimetic functions as a real antioxidant only when its product is removed rapidly by a catalase or peroxidase system. A normal cell is well equipped with multiple H_2_O_2_-removing systems such as catalases, glutathione peroxidases, glutathione transferases, peroxyredoxins and thioredoxins. Unlike many reports that have stated that an increase in ROS results in the subsequent loss of quiescence and the self-renewal properties of HSCs, our results show that MnP behaves like a mild pro-oxidant in hematopoietic cells, but increases HSPC pool and stem cell function. We next examined the redox balance of LSK cells by determining the ratio of 2x GSH/GSSG, as the glutathione redox couple is the major indicator of cellular redox state. Freshly isolated BMNCs were treated with either vehicle or 20 μM MnP for 2 h or 16 h, immunostained and flow cytometry sorted at 4 °C. Twenty thousand LSK cells were used for simultaneous analysis of GSH and GSSG by the sensitive LS/MS method we recently developed [27]. The results show no change at 2 h treatment (Fig. 5D left panel) and a slightly reduced status at 16 h with MnP *in vitro* treatment (Fig. 5D right panel). The result of the 2x GSH/GSSG ratio on MyPro cells treated with vehicle and MnP shows no difference as well (data not shown). These results support our hypothesis that the adaptive responses were able to maintain normal intracellular redox balance.

To further specify the defense capability of ROS in different lineages of the cells, we investigated the redox status of the LSK and MyPro cells. Freshly isolated, stained and flow cytometry- sorted 20,000 LSK or MyPro cells were used. The 2x GSH/GSSG ratio was 2.5x higher in LSK cells compared to that in the MyPro cells (Fig. 5E). The result suggests that LSK cells are in a more reduced status than progenitor cells are.

In the next experiment, we wanted to investigate the capacity difference of scavenging H_2_O_2_ in different lineage cells. In these experiments, the freshly isolated BMNCs cells were immunostained, incubated with H2DCFDA or DCFDA for 20 min, exposed to various concentrations of H_2_O_2_ for 15 min at 37 °C, washed, and flow cytometry analyzed. At the concentration of 50 μM H_2_O_2_, the cellular ROS increased 9.3x, 10.6x and 27x for LSK, MyPro and Lin+ cells, respectively. At 100 μM H_2_O_2_, ROS increased 14.1x, 25.3x and 49.5x for LSK, MyPro and Lin+ cells, respectively (Fig. 5F). These results further confirm that LSK cells have a higher capacity to clear H_2_O_2_ compared to mature cells. These results suggest that the transient and mild elevation of intracellular ROS caused by MnP can be overcome by the strong intracellular antioxidant defense system, especially in primitive bone marrow cells.

### 3.5 MnP induces intracellular antioxidant defenses and NRF2 signaling

**Fig. 6:**
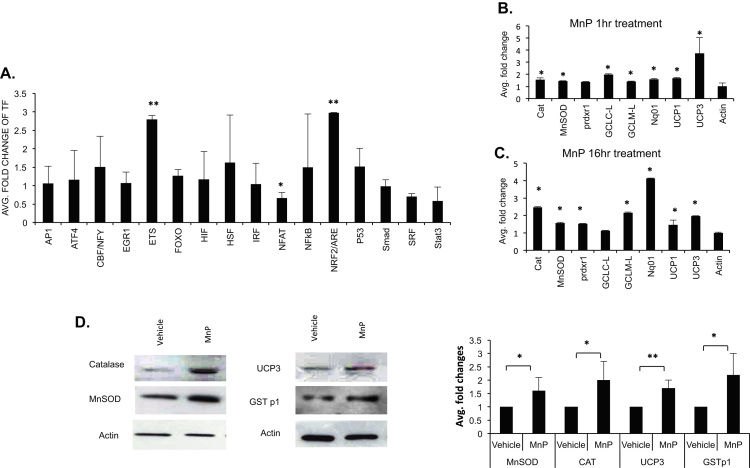
MnP increases Nrf2/ARE and ETS transcription activities. A. Oxidative transcription profiling analysis. BMNCs were isolated, treated with vehicle or 20 μM MnP for 1 h. Cells were harvested and immunostained. Nuclear extracts were isolated from flow cytometry sorted LK (lin-, ckit+) cells. Equal amounts of nuclear extracts were used for TF assay (n=3). B. Quantitative PCR on some of the Nrf2 target genes was performed on LSK cells treated with vehicle or 20 μM MnP for 1 h. C. Quantitative PCR on some of the Nrf2 target genes on LSK cells treated with vehicle or 20 μM MnP for 16 h. D. Western analysis of the protein expression of some of the Nrf2 targets. The left panel shows the representative Western blots. The right panel shows the quantitation of repeats of the experiments. The data presented are the fold changes of MnP/vehicle treated with vehicle treated as value 1. Bar graphs represent the mean+SD of at least three independent experiments except for the TF assay.

To further confirm the potential of the adaptive antioxidative mechanism and explore which pathways are affected by MnP, we performed an oxidative stress transcription factor (TF) activation profiling array. The array includes the 16 most common consensus DNA sequences of TF DNA-binding sites which respond to oxidative stress. In this experiment, BMNCs were isolated from three groups of five mice and treated with vehicle or 20 μM of MnP for 1 h *in vitro*. Triplicate nuclear extracts from 5×10^5^ flow cytometry sorted LK (Lin- and ckit +) cells were used in the assay. As shown in Fig. 6A, MnP significantly upregulated NRF2/ ARE and ETS (E twenty-six transcription factors) TF binding activities by 3 and 2.8 times, respectively, more than vehicle-treated. Nrf2 antioxidant response pathways play important roles in cellular antioxidant defense. To confirm the elevation of Nrf2 transactivation activity, we did quantitative PCR on mRNA expressions of representative Nrf2 target genes at 1 h and 16 h MnP treatments. The result shows that catalase (CAT), MnSOD, peroxiredoxin 1 (Prdx1), glutamate-L-cysteine liage (GCLC-L), glutamate-cysteine ligase regulatory subunit (GCLM-L), NAD(P)H Quinone Oxidoreductase (Nqo1), UCP1 and UCP3 were elevated at the 1 h and/or 16 h MnP *in vitro* treatments (Fig. 6B & C) respectively. Western blot analysis shows that the Nrf2 target proteins exemplified by catalase, MnSOD, GSTp1 and UCP3 were elevated (Fig. 6D). The results support our hypothesis that MnP acts as a pro-oxidant in bone marrow cells which consequently stimulates the intracellular antioxidative defense system. The results are also consistent with evidence that Nrf2 signaling plays important roles in regulating stem cell physiology [29], [30], [31]. The UCP3 elevation is consistent with our data that MnP suppresses mitochondrial function in bone marrow cells.

ETS transcription factors comprising more than 26 genes in vertebrates have been implicated in regulation of hematopoiesis. The hallmark ETS factor involved in hematopoiesis is encoded by the PU.1 gene that regulates the expression of multiple genes for different lineages during normal hematopoiesis and also directs homing and long-term engraftment of hematopoietic progenitors to bone marrow [32]. Additionally, several other ETS factors, including FLI1, TEL/ETV6 and a series of recently discovered ETS-Related Genes (ERG), have been shown to play critical roles at various levels of hematopoiesis [33]. Our oxidative gene transcription factor array shows that ETS transcription activity was elevated by MnP treatment (Fig. 6A), and our preliminary studies show that the expression of several ERG genes was altered upon MnP treatment (unpublished data). The significance of ETS target genes in the regulation of ROS is interesting and will be a topic of future investigation.

## 4 Discussion

Redox homeostasis plays pivotal roles in regulating the self-renewal, differentiation, and genomic stability of stem cells. SOD enzymes involved in dismutation of superoxide anion to hydrogen peroxide act as the first line of defense against disturbance of ROS homeostasis. Mitochondria are the main site of superoxide production. Therefore, MnSOD, located in mitochondria, is the primary antioxidant enzyme that removes superoxide radicals generated in the mitochondria. Our CAFC and CFU assays and serial BMT result show that colony forming capability and engraftments / multilineage differentiation are increased in cells treated with MnP. This observation is consistent with a recent study showing that undifferentiated embryonic stem cells (ESCs) expressed an increased amount of mitochondrial MnSOD compared to their differentiated counterpart [34]. We therefore hypothesized that SOD mimetics play roles in regulating bone marrow HSPC proliferation and differentiation through regulation of ROS.

Our observation that MnP may act as a transient pro-oxidant, as indicated by DCF and Nrf2 activation assays, is unexpected and merits careful consideration. DCF assay detects numerous reactive oxygen species including hydrogen peroxide, thiyl radicals, nitric oxide, nitrogen dioxide, and peroxynitrite [35]. At the 2 h *in vitro* treatment with MnP, we detected an increase in total ROS in all three different lineages of hematopoietic cells compared to vehicle controls. Together with our quantitation PCR and Western blots confirmation of the Nrf2 pathway activation, our results support the hypothesis that MnP acts as a mild pro-oxidant. However, in the 60-day MnP *in vivo-* treated and MnSOD transgenic bone marrow cells, we did not detect ROS change. Furthermore, we did not detect any significant changes in the GSH/GSSG redox couple on MnP- treated LSKs at 2 h and 16 h. These results suggest that the ROS increase in the *in vitro-* treated cells did not disturb the GSH/GSSG redox homeostasis. Our examinations of the antioxidant capacity of different lineage cells show clearly that the more primitive LSK cells are in a more reduced physiological status and have a much stronger capacity to clear H_2_O_2_ compared to more mature cells. This evidence strengthens our hypothesis that, acting as a mild pro-oxidant, MnP activates such endogenous oxidative defense systems as the Nrf2 pathway, thereby not only regulating intracellular redox homeostasis but possibly also affecting bone marrow HSPC regulation.

**Fig. 7:**
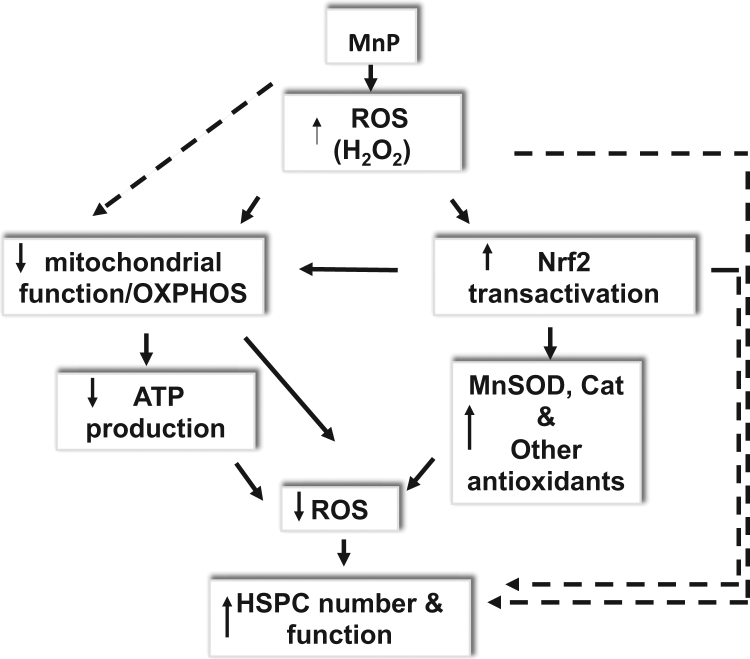
A schematic hypothesis of the mechanism of bone marrow HSPCs enhancement by MnP.

Nrf2 signaling is one of the best-characterized defense mechanisms that acts against oxidative insults. It controls the basal and induced expression of an array of antioxidant genes that regulate physiological and pathophysiological oxidative responses. Evidence shows that Nrf2 also plays a pivotal role in regulation of stem cells. A recent report shows that Nrf2 is highly expressed in undifferentiated HSCs compared with differentiated cells. The same study also shows that a reduction of Nrf2 activity enhances the differentiation of HSCs and that the reversal of Nrf2 knockdown increases pluripotency [29]. Despite the function of transactivation of downstream antioxidant genes, the role of Nrf2 on HSC maintenance can also be independent of cellular ROS concentration [30]. As reported, Nrf2 directly binds to ARE in the promoter region of Cxcr4, which is essential for the maintenance of the quiescent HSC pool [31]. Thus, it is possible that MnP, as a mild pro-oxidant and a ROS regulator, activates the intracellular oxidative defense system through Nrf2 signaling, leading to Nrf2- dependent and -independent enhancement of bone marrow stem cells, as illustrated in Fig. 7.

It has been suggested that different types of ROS molecules might provide a fine mechanism for tuning redox signaling to control the delicate balance among cell growth, proliferation, and differentiation of HSCs [36]. A reduction in MnSOD activity favors proliferation of MEFs, while induction of MnSOD activity facilitates a proliferation of cells transitioning into quiescence [37]. MnSOD -/- MEFs failed to exit from the proliferative cycle and showed increased cyclin D1 and cyclin B1 accumulation compared to MnSOD +/+ MEFs. Overexpression of MnSOD in MnSOD +/- MEFs suppresses O_2_^−.^, decreases cyclin B1, and facilitates cell transit into quiescence. Our finding is in agreement with the hypothesis that MnSOD, by catalyzing O_2_^-.^ to H_2_O_2_, switches the superoxide-signaling pathway that regulates proliferation to a hydrogen peroxide-signaling pathway that supports quiescence, demonstrating that the roles and functions of ROS in stem cells are diverse and context dependent.

A recent report demonstrates that under oxidative stress, increased ROS production is counteracted by Nrf2-dependent transcriptional upregulation of mitochondrial uncoupling protein 3, UCP3, *via* Nrf2 binding to the ARE site on the UCP3 promoter [38]. H_2_O_2_ increases the UCP3-mediated proton leak, and consequently decreases the production of superoxide from the electron transport chain [39]. Nrf2 is also known to be an important player in the maintenance of mitochondrial homeostasis through regulation of mitochondrial membrane potential, OXPHOS and ATP production [40]. The reports are in agreement with our data showing that MnP increases UCPs through elevated Nrf2 signaling and, in turn or independently, decreases mitochondrial function that further reduces endogenously generated ROS.

SOD mimetics, by their structures, act more as redox regulators than simple antioxidants. They can be an oxidant or a reductant inside cells and have an antioxidative impact only if H_2_O_2_ is efficiently removed [18].

(H_2_O)_2_Mn^III^P^5+^ +O_2_^·-^ <===> (H_2_O)Mn^II^P^4+^ +O_2_ +H_2_O, k_red_(O_2_^·-^)

(H_2_O_2_)Mn^II^P^4+^ +H_2_O+2H^+^ +O_2_^·-^ <===> (H_2_O)_2_Mn^III^P^5+^ + H_2_O_2_, k_ox_(O_2_^·-^)

This feature gives them the potential to leverage the physiological properties of cells that have different antioxidant capacities to regulate intracellular ROS homeostasis. As mentioned above, SOD mimetics not only have been reported to provide radioprotection in animal models, they have been shown to be radio- and chemo- sensitizers, as they radio- sensitize treatments in head and neck tumors in mice [19], brain glioma mouse model [41] and a 4T1 mouse breast cancer xenograft model [42].

It is worth mentioning that Weimin Miao and his colleagues have recently reported that overexpression of MnSOD by retroviral transduction exhibited an insignificant effect on long-term engraftment of transplanted HSCs. However, overexpression of catalase has a significant beneficial effect [43]. In their paper, the authors report that the product of MnSOD, H_2_O_2_, can act as a pro-oxidant and therefore could be damaging stem cells unless the cells were co-transfected with catalase to remove H_2_O_2_. Indeed, the result is in agreement with our data that MnP acting as a pro-oxidant increases Nrf2-mediated up regulation of catalase and other antioxidative enzymes. The adaptive effect could be the reason that stem cell function was enhanced in our study. In the same study, the authors also demonstrated that administration of a MnSOD plasmid and lipofectin complex (MnSOD-PL) is protective against radiation-induced injury of stem cells without concurrent overexpression of catalase. This finding also supports the radio-protective effect of MnP reported in other studies.

Our results suggest that MnP in bone marrow cells reduces O2^•-^ to H_2_O_2_. The elevated H_2_O_2_ activates intracellular Nrf2 signaling, which activates the production of antioxidant enzymes. MnP through Nrf2-dependent up-regulation of UCPs or independently reduces mitochondrial function and ATP synthesis. The adaptive responses of both decreased mitochondrial OXPHOS and increased intracellular antioxidant defenses lead to a concomitant decrease in ROS levels, which contributes to the improvement of stem cell maintenance. In addition, MnP, *via* an Nrf2-independent regulation of stem cells and activation of the ETS system, may also contribute to the increase in stem cell production and function (Fig. 7).

## 5 Conclusions

This study is the first to report two novel findings: A mild pro-oxidant could induce stem cell function through adaptive responses that include suppression of mitochondrial respiration and stimulation of endogenous antioxidant defenses. The bone marrow stem cells and early progenitor cells have a higher capacity to remove H_2_O_2_ than mature cells have. MnP has been used in clinical trials for its radiation-protection during cancer treatment. It has been reported that MnP acts as a sensitizer for cancer treatment in animals. The finding that MnP enhances the LSK cell pool augments the application of MnP as a potential novel agent clinically. Importantly, our results contribute to a better understanding of stem cell regulation by ROS. Numerous studies in the past decade have uncovered the importance of redox signaling to the biology of stem cells. However, the differential effect of different ROS molecules on stem cell regulation remains unclear and warrants further in-depth studies. It also would be interesting to investigate further the properties of MnP on bone marrow stem cells under pathological conditions where replenishment of hematopoietic cells is urgently needed.

## Appendix A Supplementary material

Supplementary material:**Supplement 1. MnP treatment does not affect mature bone marrow cells by serial bone marrow transplantations.** A. Donor cells were isolated from 60-day vehicle or MnP *in vivo-* treated mice. B. The donor cells were *in vitro* treated with vehicle or 20 μM MnP (MnP) for 16 h. Percentage of donor derived Granulocyte, T-cell, B-cell and total cell engraftment (Ly5.2 positive) at wk 48 post the 1st BMT were analyzed by flow cytometry and presented (n=6 for each treatment).

## Footnotes

Appendix A Supplementary data associated with this article can be found in the online version at doi:10.1016/j.redox.2017.02.005.
