# Supplementary figures and images for: A novel redox regulator, MnTnBuOE-2-PyP5+, enhances normal hematopoietic stem/progenitor cell function
Source: Redox Biol. 2017 Feb 10;12:129–38. doi: 10.1016/j.redox.2017.02.005 (PMC5320058; doi:10.1016/j.redox.2017.02.005)

# Supplement 1.

A.

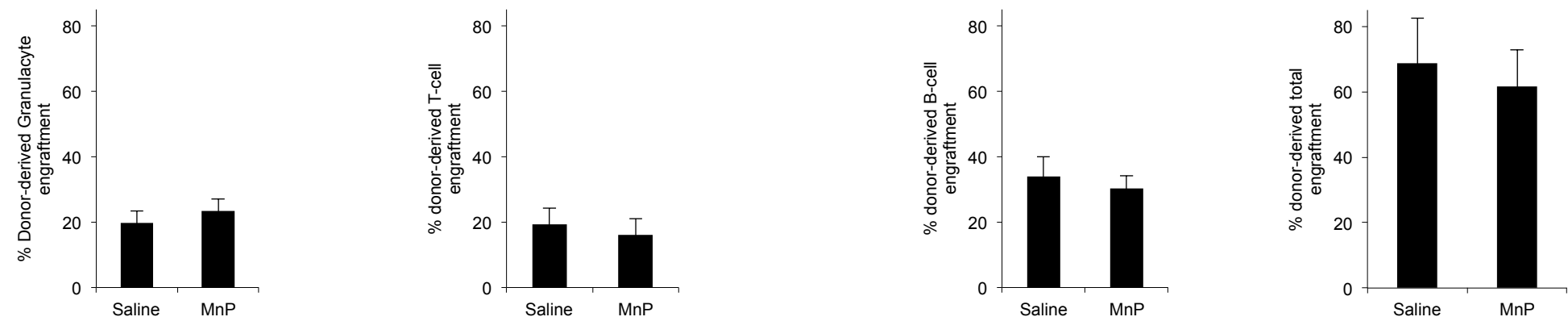

B.

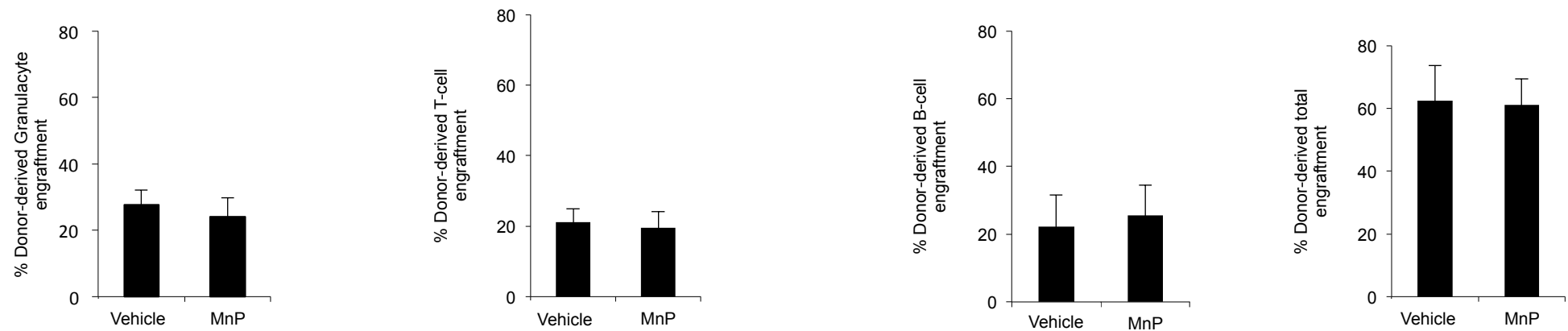

Supplement: Supplementary file 1 — Supplementary material:Supplement 1. MnP treatment does not affect mature bone marrow cells by serial bone marrow transplantations. A. Donor cells were isolated from 60-day vehicle or MnP in vivo- treated mice. B. The donor cells were in vitro treated with vehicle or 20 μM MnP (MnP) for 16 h. Percentage of donor derived Granulocyte, T-cell, B-cell and total cell engraftment (Ly5.2 positive) at wk 48 post the 1st BMT were analyzed by flow cytometry and presented (n=6 for each treatment). [file mmc1.pdf]
